# Beauvericin inhibits melanogenesis by regulating cAMP/PKA/CREB and LXR-α/p38 MAPK–mediated pathways

**DOI:** 10.1038/s41598-018-33352-8

**Published:** 2018-10-08

**Authors:** Seung Eun Lee, See-Hyoung Park, Sae Woong Oh, Ju Ah Yoo, Kitae Kwon, Se Jung Park, Jangsoon Kim, Hak Sung Lee, Jae Youl Cho, Jongsung Lee

**Affiliations:** 1 Molecular Dermatology Laboratory, Department of Integrative Biotechnology, College of Biotechnology and Bioengineering, Sungkyunkwan University, Suwon City, 16419 Gyunggi Do Republic of Korea; 2 Molecular Immunology Laboratory, Department of Integrative Biotechnology, College of Biotechnology and Bioengineering, Sungkyunkwan University, Suwon City, 16419 Gyunggi Do Republic of Korea; 3 Biocosmetics Research Center, College of Biotechnology and Bioengineering, Sungkyunkwan University, Suwon City, 16419 Gyunggi Do Republic of Korea; 4 Department of Bio and Chemical Engineering, Hongik University, 30016 Sejong City, Republic of Korea; 5 Food Science R&D Center, Kolmar BNH Co., Ltd., 30003 Sejong City, Republic of Korea

## Abstract

Melanogenesis is the process of production of melanin pigments that are responsible for the colors of skin, eye, and hair and provide protection from ultraviolet radiation. However, excessive levels of melanin formation cause hyperpigmentation disorders such as freckles, melasma, and age spots. Liver X receptors (LXR) are nuclear oxysterol receptors belonging to the family of ligand-activated transcription factors and physiological regulators of lipid and cholesterol metabolism. In the skin, activation of LXRs stimulates differentiation of keratinocytes and augments lipid synthesis in sebocytes. However, the function of LXRs in melanogenesis has not been clearly elucidated. In addition, although beauvericin, a well-known mycotoxin primarily isolated from several fungi, has various biological properties, its involvement in melanogenesis has not been reported. Therefore, in this study, we examined the effects of beauvericin on melanogenesis and its molecular mechanisms. Beauvericin decreased melanin content and tyrosinase activity without any cytotoxicity. Beauvericin also reduced protein levels of MITF, tyrosinase, TRP1, and TRP2. In addition, beauvericin suppressed cAMP-PKA-CREB signaling and upregulated expression of LXR-α, resulting in the suppression of p38 MAPK. Our results indicate that beauvericin attenuates melanogenesis by regulating both cAMP/PKA/CREB and LXR-α/p38 MAPK pathways, consequently leading to a reduction of melanin levels.

## Introduction

Melanogenesis is the production of melanin pigments in melanosomes by melanocytes, which are distributed in the basal layer of the epidermis^1^. Melanin is responsible for the color of skin, eye, and hair and has an important protective function against ultraviolet (UV) radiation^2^. However, overproduction and accumulation of melanin results in hyperpigmented disorders, including freckles, melasma, and age spots^3^. Therefore, melanogenesis should be properly regulated to maintain a healthy skin condition. For this reason, there is an increasing need for regulators of melanogenesis to treat pigmented skin diseases.

A number of signaling pathways are known to regulate melanogenesis. First, cyclic AMP leads to phosphorylation of CREB transcription factor and then stimulates expression of the microphthalmia transcription factor (*MITF*) gene^1,4^. MITF is a transcription factor that binds to the promoter region of the melanogenic genes tyrosinase-related protein (TRP)1, TRP2, and tyrosinase and upregulates their expression^1,5^. Tyrosinase produces melanins and TRPs are important in the regulation of melanin synthesis^5^. Expression of the *MITF* gene is regulated by several signaling pathways. JNK and p38 mitogen-activated protein kinase (MAPK) are involved in activation of MITF expression and the consequent increased tyrosinase expression^6^. ERK activation signals also increase CREB phosphorylation and subsequent MITF expression^7,8^. In addition, melanogenesis is upregulated by the NF-κB signaling pathway^9,10^. In contrast, melanogenesis is inhibited through the liver X receptor (LXR)-mediated pathway^11^.

Liver X Receptors (LXRs) are nuclear oxysterol receptors belonging to the family of ligand-activated transcription factors^12^ and physiological regulators of lipid and cholesterol metabolism^13,14^. In addition, LXRs are involved in the inflammatory response and immunity^15^. LXRs have two isoforms: LXR-α, which is highly expressed in the liver and adipose tissue, and LXR-β, which is more ubiquitous^16^. In addition, LXR-α is expressed and plays an important role in the skin^17^. In particular, LXR-α expression is relatively high in perilesional melanocytes^18,19^ and is involved in regulation of melanogenesis^20^.

**Figure 1:**
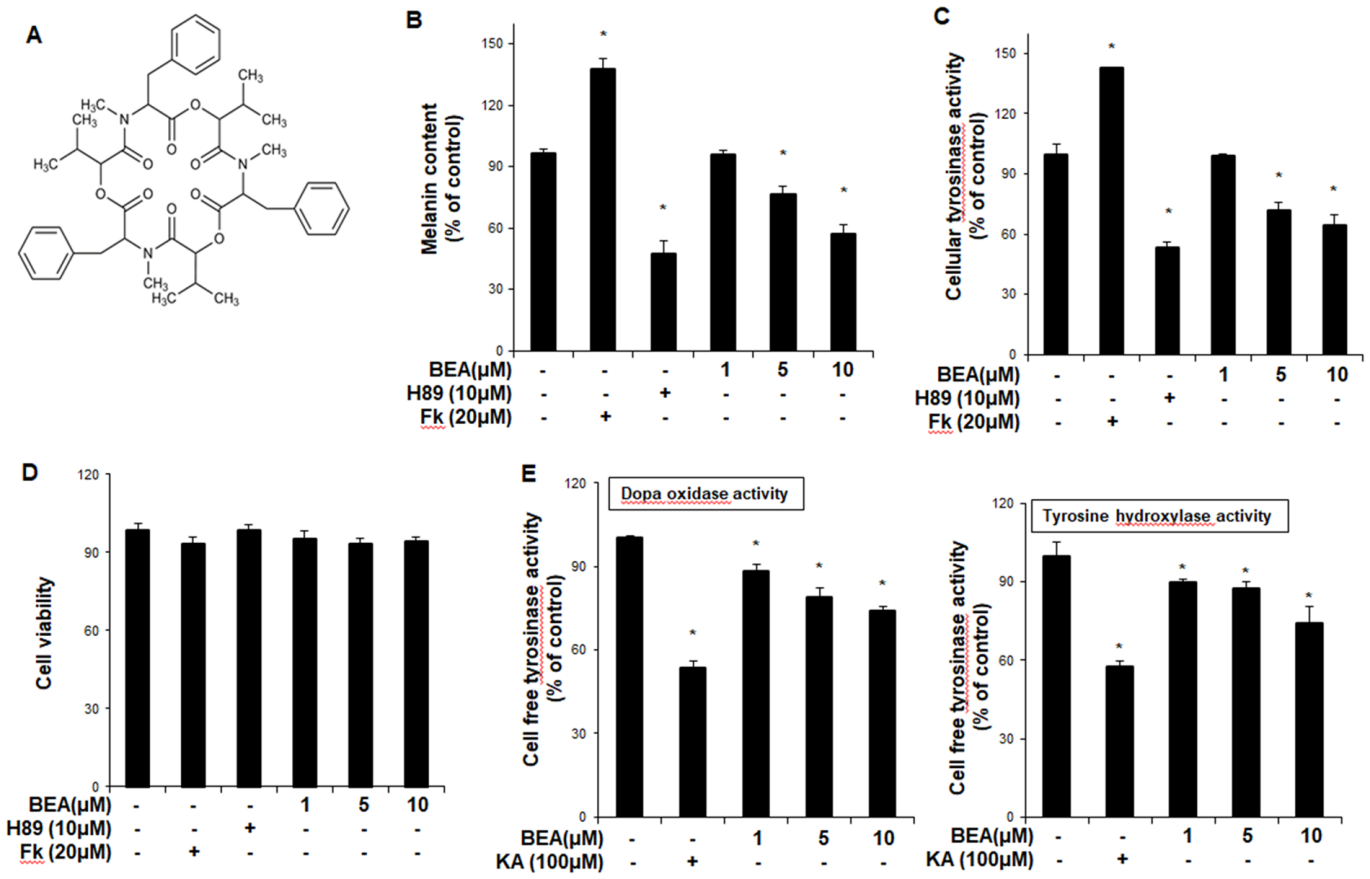
The anti-melanogenic effect of beauvericin in B16F10 cells. (**A**) Chemical structure of beauvericin. (**B**) B16F10 cells were treated with beauvericin for 24 h. After harvesting, the cells were dissolved in a mixture of Soluene-350 and water. The melanin content was measured by absorbance at 500 nm (**B**). (**C**) B16F10 cells were treated with beauvericin for 24 h. After harvesting, the cells were lysed by sonication and assayed for cellular tyrosinase activity (dopa oxidase). Absorbance was immediately measured at 505 nm. Results were confirmed from at least three independent experiments, and values represent the means ± SEM. ^*^P < 0.05 vs. untreated control. (**D**) Cell counting kit-8 was used to assay cell viability. Results were confirmed from at least three independent experiments, and values represent the means ± SEM. ^*^P < 0.05 versus untreated control. (**E**) Cellular tyrosinase was isolated from B16F10 melanoma cells. After protein quantification, dopa oxidase activity and tyrosine hydroxylase activity activities were performed. The absorbance was measured spectrophotometrically at 505 nm and 475 nm, respectively. Data are presented as the means ± SEM of four independent experiments. Statistical significance of differences among the groups were assessed using the one-way analysis of variance (ANOVA) test followed by Tukey’s multiple-comparison test in the GraphPad Prism 5 Software. *P < 0.05 vs. control group. Fk, forskolin; KA: kojic acid; BEA, beauvericin.

Beauvericin, an important bioactive metabolite of *Cordyceps* species, is a well-known mycotoxin that is primarily isolated from several fungi, such as an entomopathogenic fungus *Beauveria bassiana* and *Fusarium* spp. Beauvericin belongs to the enniatin antibiotic family and its structure is a cyclic hexadepsipeptide that contains three D-hydroxyisovaleric acid residues alternating with three N-methylphenylalanine residues (Fig. 1A). Beauvericin has been reported to have several properties such as insecticidal, antimicrobial, and anti-tumor activities^21,22^. It exerts its anti-cancer effects by inducing apoptosis in several cancer cells including CCRF-CEM leukemia cells, human non-small cell lung cancer A549 cells, human colon adenocarcinoma Caco-2 cells, and H4IIE hepatoma cells^23–25^. Beauvericin is also known to have an ionophoric property and regulates translocation of specific ions through cellular channels^26–30^. In addition, beauvericin has anti-inflammatory effects by inhibiting T cell activation through downregulation of the PI3K/Akt signaling pathway^31^. Although these various properties of beauvericin are well recognized, its effects on skin biology have not been reported.

In this study, we examined the effects of beauvericin on melanogenesis and its molecular mechanisms.

## Results

### Beauvericin inhibits melanogenesis in B16F10 cells and human epidermal melanocytes

**Figure 2:**
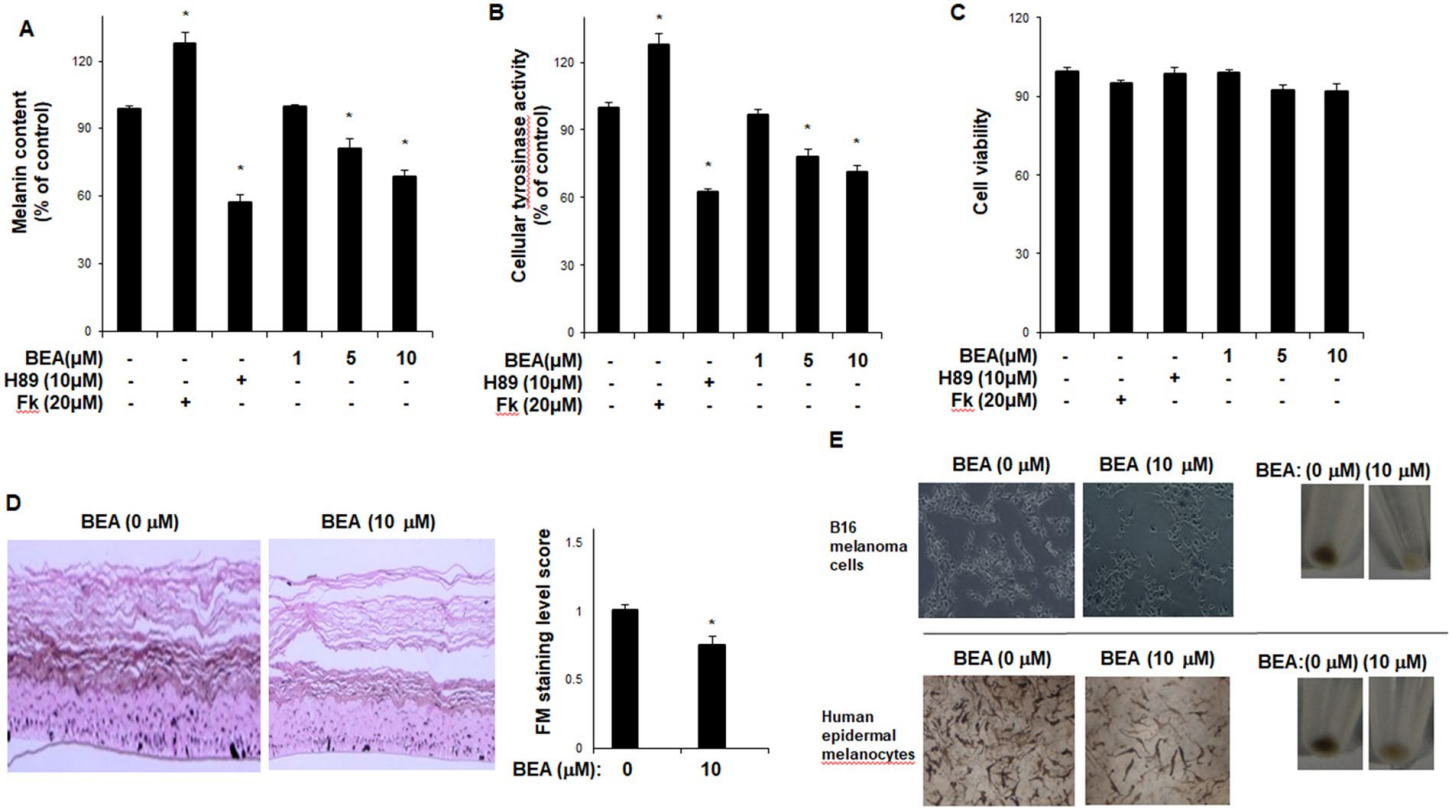
The inhibitory effect of beauvericn on melanogenesis in human epidermal melanocytes. (**A**) Human epidermal melanocytes were treated with beauvericin for 48 h. After harvesting, the cells were dissolved in a mixture of Soluene-350 and water. The melanin content was measured by absorbance at 500 nm. *P*-values were obtained by the one-way ANOVA. (**B**) Human epidermal melanocytes were treated with beauvericin for 48 h. After harvesting, the cells were lysed by sonication and assayed for cellular tyrosinase activity (dopa oxidase). Absorbance was immediately measured at 505 nm. Results were confirmed from at least three independent experiments, and values represent the means ± SEM. *P < 0.05 vs. untreated control. *P*-values were obtained by the one-way ANOVA. (**C**) Cell counting kit-8 was used to assay cell viability. Results were confirmed from at least three independent experiments, and values represent the means ± SEM. *P < 0.05 versus untreated control. *P*-values were obtained by the one-way ANOVA. (**D**) Reconstructed epidermis was incubated with beauvericin (10 μM) for 3 days. The epidermis was subjected to Fontana–Masson staining. Quantification of Fontana-Masson staining was performed using Image J. Data are presented as the means ± SEM of three independent experiments. *P < 0.05 vs. control group. *P*-values were obtained by Student’s *t*-test. (**E**) After B16F10 cells and human epidermal melanocytes were incubated with the indicated concentration of beauvericin for 24 h or 48 h, imaging analysis was performed. Fk, forskolin; BEA, beauvericin.

To examine the effects of beauvericin on melanogenesis, a melanin content assay was performed in mouse melanoma B16F10 cells. As shown in Fig. 1B, beauvericin reduced melanin content in a concentration-dependent manner. In addition, cellular tyrosinase activity was suppressed by beauvericin treatment (Fig. 1C). In these experiments, H89, a specific inhibitor of PKA, and forskolin were used as a negative control and a positive control, respectively. As shown in Fig. 1B,C, forskolin significantly increased melanin content and cellular tyrosinase activity, while H89 significantly reduced them. We found that beauvericin showed no cytotoxic effects at the concentrations tested (Fig. 1D). In a cell-free tyrosinase activity assay, beauvericin suppressed tyrosinase activity (tyrosine hydroxylase and dopa oxidase) concentration-dependently (Fig. 1E). Consistent with these findings, we found that beauvericin reduced melanin content (Fig. 2A) and cellular tyrosinase activity (Fig. 2B) in human epidermal melanocytes. As shown in Fig. 2A,B, while forskolin significantly increased melanin content and cellular tyrosinase activity, H89 significantly reduced them. We also found that beauvericin showed no cytotoxicity at the concentrations treated (Fig. 2C). In addition, using Fontana-Mason staining, we found that treatment with beauvericin reduced the level of melanin in reconstructed epidermis (Fig. 2D). The imaging results of B16F10 cells and human epidermal melanocytes treated with beauvericin are shown in Fig. 2E. These data indicate that beauvericin has anti-melanogenic activity, and suggest that its effects may be mediated by regulating the expression of melanogenic genes as well as by suppressing tyrosinase activity.

### Beauvericin regulates expression of melanogenic genes

**Figure 3:**
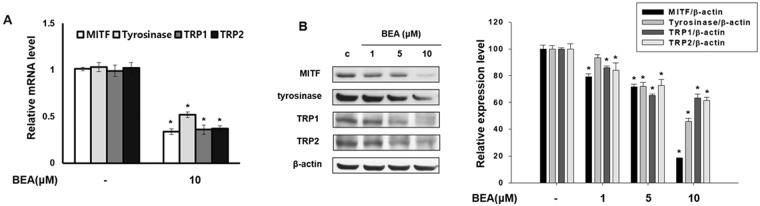
Beauvericin inhibits the expression of melanogenesis-related genes. (**A**) B16F10 cells were incubated with the indicated concentrations of beauvericin for 24 h. mRNA levels of melanogenesis-related genes MITF, tyrosinase, TRP-1, and TRP-2 were determined by real-time PCR. The results were expressed relative to untreated cells after normalization against the GAPDH level. Results were confirmed from at least three independent experiments and values represent the means ± SEM. *P < 0.05 vs. untreated control. *P*-values were obtained by Student’s *t*-test. (**B**) B16F10 cells were incubated with the indicated concentrations of beauvericin for 24 h. The protein levels of MITF, tyrosinase, TRP-1, and TRP-2 were determined by Western blot analysis. Equivalent amounts of protein were analyzed. Results were confirmed from at least three independent experiments and values represent the means ± SEM. *P*-values were obtained by the one-way ANOVA. *P < 0.05 vs. untreated control. BEA, beauvericin.

To investigate the effects of beauvericin on expression of melanogenesis-related genes including MITF, tyrosinase, TRP1, and TRP2, Western blot and real-time PCR analyses were performed. Beauvericin decreased mRNA levels of MITF, tyrosinase, TRP1, and TRP2 (Fig. 3A). In addition, the levels of these proteins were also decreased by beauvericin treatment (Fig. 3B). These results indicate that anti-melanogenic effects of beauvericin are mediated by downregulating the expression of melanogenic genes.

### Inhibitory effect of beauvericin on cAMP-related signaling pathway

**Figure 4:**
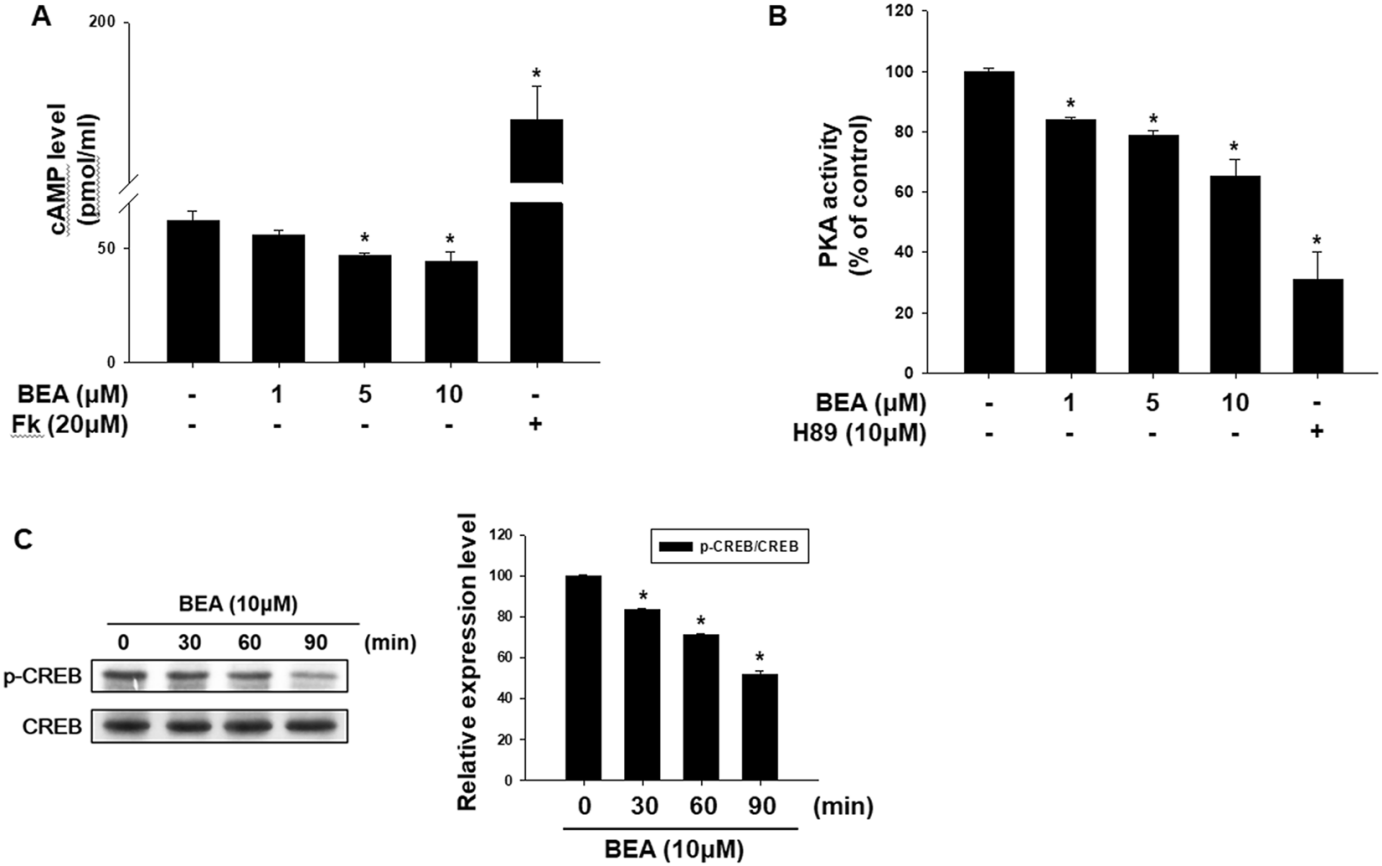
Suppression of cAMP-related signaling in response to treatment with beauvericin. (**A**) Intracellular cAMP levels were analyzed using a cAMP assay ELISA kit. Cells were treated with the indicated concentration of beauvericin for 4 h. Results were confirmed from at least three independent experiments and values represent the means ± SEM. *P*-values were obtained by the one-way ANOVA. *P < 0.05 vs. untreated control. (**B**) Activity of PKA, the downstream molecule of cAMP, was assayed using an ELISA kit. Cells were treated with the indicated concentration of beauvericin for 4 h. Results were confirmed from at least three independent experiments and values represent the means ± SEM. *P*-values were obtained by the one-way ANOVA. *P < 0.05 vs. untreated control. (**C**) Phosphorylation levels of CREB were determined by Western blot analysis. Cells were treated with 10 μM beauvericn for the indicated time and the level of CREB phosphorylation was analyzed. Equivalent amounts of protein were analyzed for each condition. Results were confirmed from at least three independent experiments and values represent the means ± SEM. *P*-values were obtained by the one-way ANOVA. *P < 0.05 vs. untreated control. FK, forskolin; BEA, beauvericin.

We found that beauvericin inhibits melanogenesis through the downregulation of MITF, consequently inhibiting expression of tyrosinase, TRP1, and TRP2. It is well known that the cAMP signaling pathway contributes to melanogenesis^32–34^. Therefore, to examine the involvement of cAMP signaling in the anti-melanogenesis activity of beauvericin, we performed ELISA for protein kinase A (PKA) activity and cAMP and Western blot analysis for phosphorylated CREB. As shown in Fig. 4A, cAMP production was reduced by beauvericin treatment. Activity of PKA (a cAMP-dependent molecule) was also suppressed (Fig. 4B). As expected, beauvericin reduced phosphorylation of CREB, a substrate of PKA (Fig. 3C). These data indicate that beauvericin inhibits melanogenesis by suppressing cAMP-PKA-CREB signaling.

### Involvement of MAPKs and NF-κB in the anti-melanogenesis effect of beauvericin

**Figure 5:**
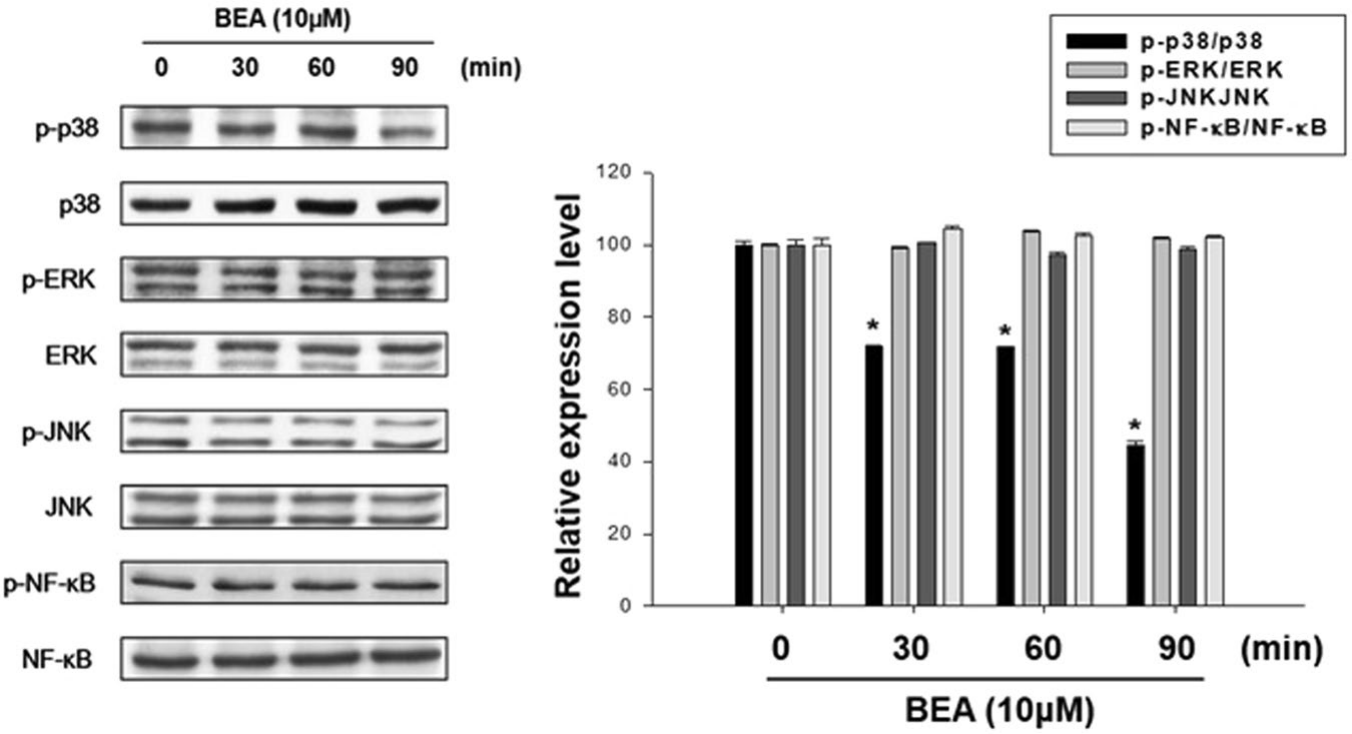
Effects of beauvericin on phosphorylation of MAPKs and NF-κB in B16F10 cells. Cells were treated with 10 μM beauvericn for the indicated time and the phosphorylation levels of proteins were determined by Western blot analysis. Results were confirmed from at least three independent experiments and values represent the means ± SEM. *P*-values were obtained by the one-way ANOVA. *P < 0.05 vs. untreated control. p38, p38 MAPK; BEA, beauvericin.

To examine the involvement of MAPKs and NF-κB in the beauvericin-induced inhibition of melanogenesis, Western blot analysis was performed to measure the phosphorylated levels of MAPKs and NF-κB. As shown in Fig. 5, beauvericin reduced the phosphorylated levels of p38 MAPK, but had no effects on phosphorylated levels of NF-κB, ERK, and JNK. These results suggest that beauvericin suppresses melanogenesis by inhibiting the p38 MAPK signaling pathway.

### Involvement of liver X receptor-α (LXR-α) in beauvericin-induced depigmentation

**Figure 6:**
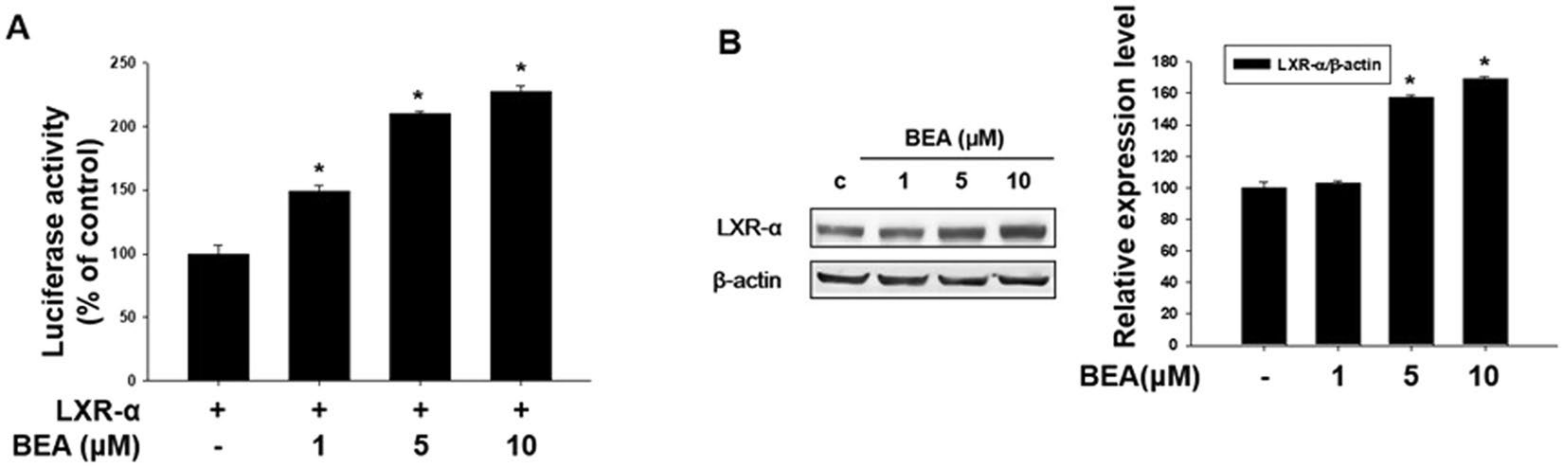
Beauvericin upregulates the expression of liver X receptor-α (LXR-α). (**A**) B16F10 cells were co-transfected with the liver X receptor (LXR)-α promoter-luciferase reporter and β-galactosidase reporter vector using PEI transfection reagent. After 24 h, transfected cells were incubated with the indicated concentrations of beauvericin. LXR-α promoter-luciferase reporter activity was determined. β-galactosidase assay was used to measure transfection efficiency. Results were confirmed from at least three independent experiments and values represent the means ± SEM. *P*-values were obtained by the one-way ANOVA. *P < 0.05 vs. untreated control. (**B**) Cells were treated with the indicated concentrations of beauvericin for 24 h. Cells were harvested, lysed, and the protein level of LXR-α was examined by Western blot analysis. Results were confirmed from at least three independent experiments, and the values represent the means ± SEM. *P*-values were obtained by the one-way ANOVA. *P < 0.05 versus untreated control. BEA, beauvericin.

In this study, we examined whether beauvericin-induced anti-melanogenesis was related to expression of the LXR-α gene. First, we investigated the effect of beauvericin on the liver X receptor (LXR)-α promoter-luciferase reporter activity. As shown in Fig. 6A, LXR-α promoter-luciferase reporter activity assay showed that LXR-α promoter-luciferase reporter activity was significantly increased following beauvericin treatment in a concentration-dependent manner. In addition, we examined the protein level of LXR-α using Western blot assay. The protein expression of LXR-α was upregulated by beauvericin in a concentration-dependent manner (Fig. 6B). These results suggest that the anti-melanogenic effect of beauvericin is related to the LXR-α signaling pathway.

**Figure 7:**
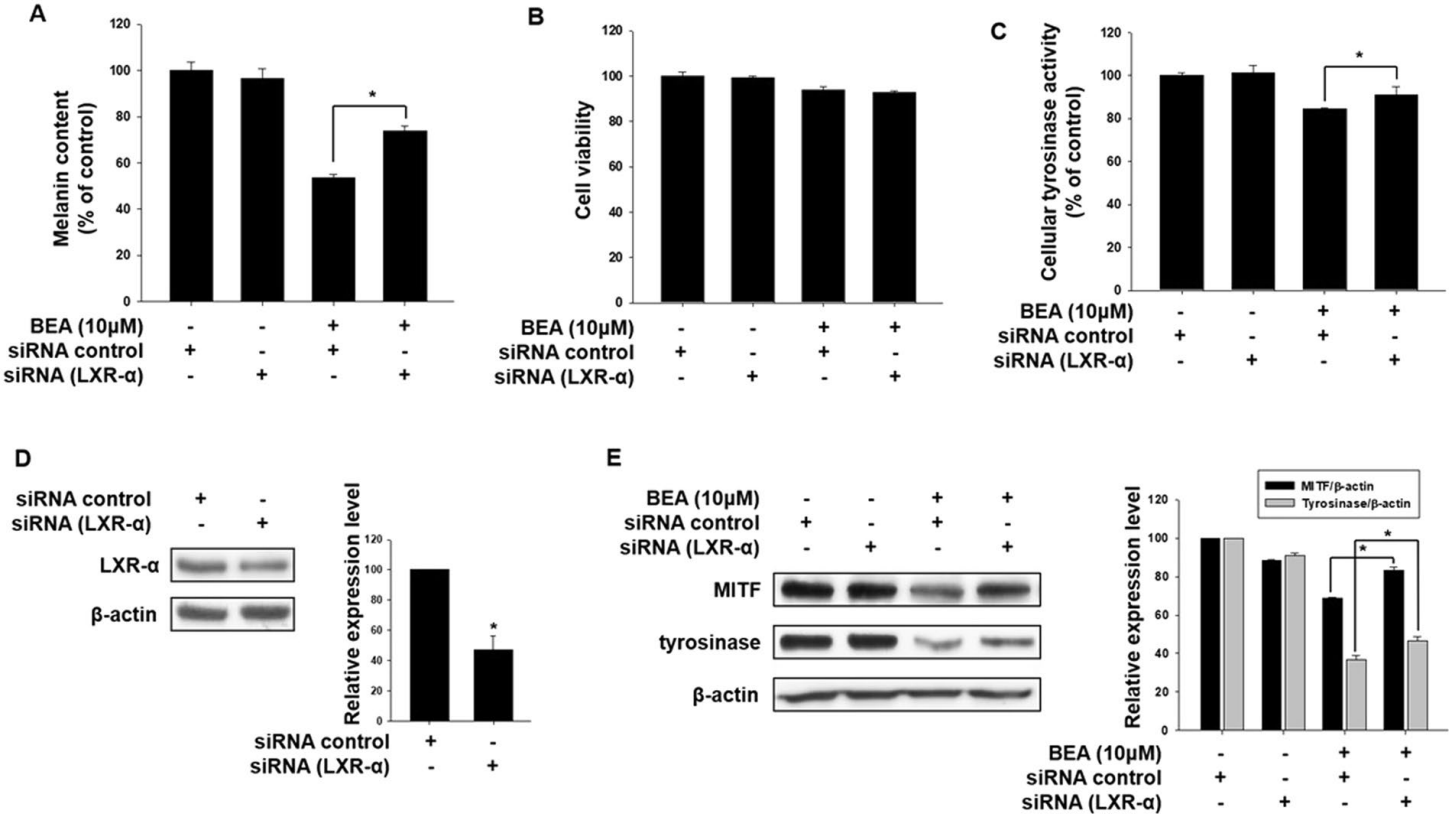
LXR-α promotes inhibition of melanogenesis by beauvericin. (**A–C**) B16F10 cells were transfected with LXR-α siRNA and incubated for 16 h. Transfected cells were treated with 10 μM beauvericin and further incubated for 24 h. Cells were harvested and assayed for melanin content (**A**) cell viability (**B**) and cellular tyrosinase activity (**C**). Results were confirmed from at least three independent experiments and values represent the means ± SEM. *P*-values were obtained by the one-way ANOVA. *P < 0.05 vs. beauvericin-treated control. (**D**) The expression level of LXR-α gene was measured by Western blot analysis to confirm the efficacy of LXR-α siRNA. B16F10 cells were transfected with LXR-α siRNA and incubated for 16 h. After transfection, the cells were further incubated for 24 h and then harvested and subjected to Western blot analysis for LXR-α. Results were confirmed from at least three independent experiments and values represent the means ± SEM. *P*-values were obtained by Student’s *t*-test. *P < 0.05 vs. untreated control. (**E**) Effects of LXR-α siRNA transfection on the beauvericin-induced reduction of protein levels of MITF and tyrosinase. B16F10 cells were transfected with LXR-α siRNA and incubated for 16 h. Transfected cells were treated with 10 μM beauvericin and further incubated for 24 h before Western blot analysis. In addition densitometric analysis was performed. Results were confirmed from at least three independent experiments. *P*-values were obtained by the one-way ANOVA. BEA, beauvericin.

To further confirm involvement of LXR-α in the anti-melanogenic effects of beauvericin, melanin content and cell viability assays were performed in the presence of LXR-α siRNA. As shown in Fig. 7A, the anti-melanogenic effects of beauvericin were attenuated by transfection with siRNA specific for LXR-α, indicating that beauvericin inhibits melanogenesis by upregulating expression of the LXR-α gene. In this experiment, beauvericin and siRNA transfection showed no cytotoxic effects (Fig. 7B). Similar to the melanin content assay, the reduced cellular tyrosinase activity induced by beauvericin treatment was recovered by LXR-α siRNA transfection (Fig. 7C). We also confirmed the efficacy of the LXR-α siRNA used in these experiments. As shown in Fig. 7D, the protein level of LXR-α was significantly reduced by transfection of LXR-α siRNA. In addition, we examined the effect of LXR-α siRNA transfection on protein levels of MITF and tyrosinase. As expected, the inhibitory effects of beauvericin on expressions of MITF and tyrosinase genes were significantly attenuated by LXR-α siRNA transfection (Fig. 7E). These data indicate that upregulation of LXR-α gene contributes to the effects of beauvericn on melanogenesis.

### Effect of liver X receptor-αagonist TO901317 on p38 MAPK phosphorylation

**Figure 8:**
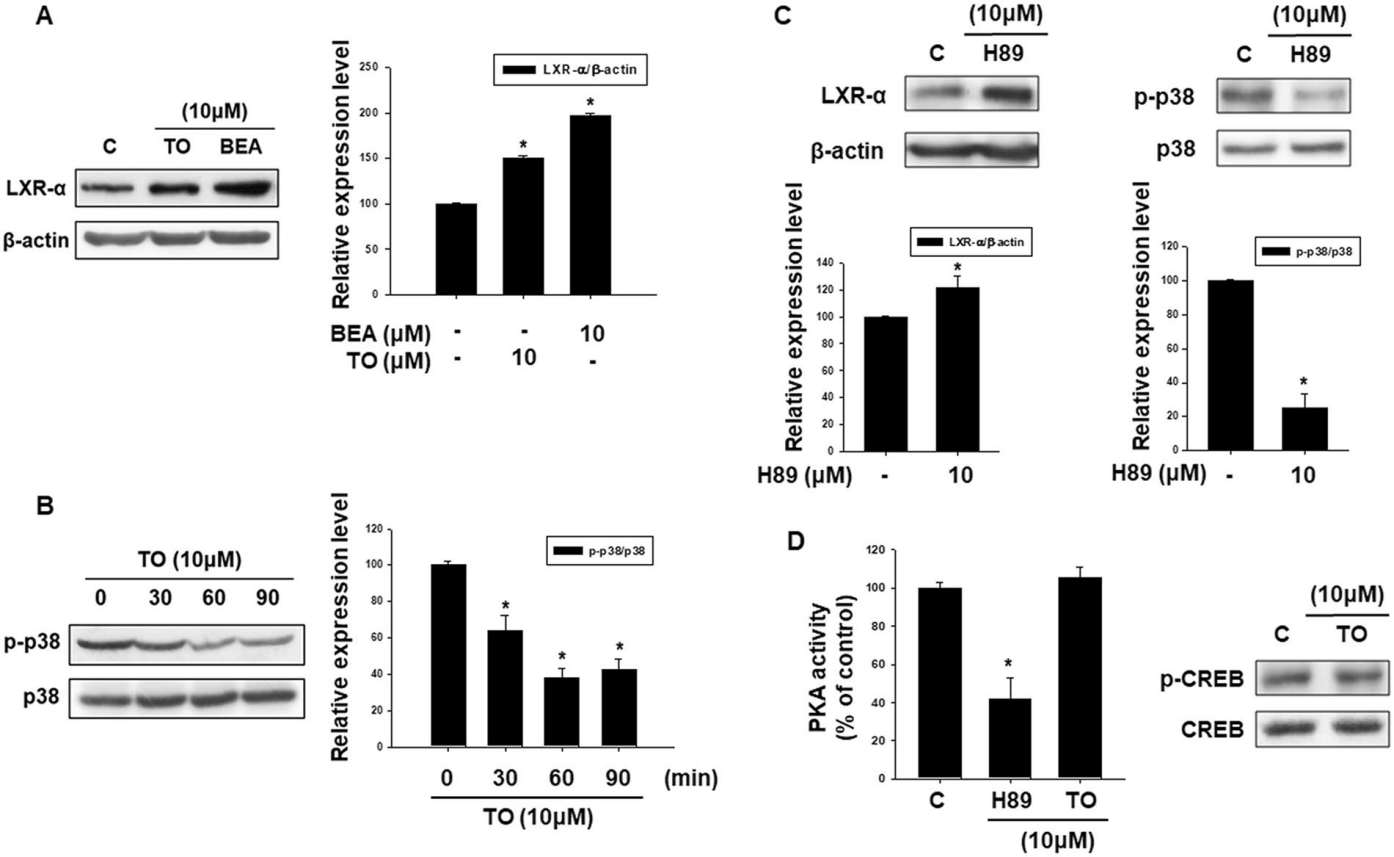
The role of LXR-α and p38 MAPK in beauvericin-induced anti-melanogenesis. (**A**) To confirm the action of TO901317, a LXR-α agonist, the expression level of LXR-α was examined by Western blotting. B16F10 cells were treated with TO901317 and beauvericin for 24 h and equivalent amounts of protein were assayed. Results were confirmed from at least three independent experiments and values represent the means ± SEM. *P*-values were obtained by the one-way ANOVA. *P < 0.05 vs. untreated control. (**B**) Phosphorylation of p38 MAPK by LXR-α agonist TO901317 was analyzed by Western blot. B16F10 cells were treated with TO901317 for the indicated time and harvested. Equivalent amounts of protein were assayed by Western blot. Results were confirmed from at least three independent experiments and values represent the means ± SEM. *P*-values were obtained by the one-way ANOVA. *P < 0.05 vs. untreated control. (**C**) Protein level of LXR-α and phosphorylation level of p38 MAPK were examined by Western blot analysis after treatment with the PKA inhibitor H89. Results were confirmed from at least three independent experiments and values represent the means ± SEM. *P*-values were obtained by Student’s *t*-test. *P < 0.05 vs. untreated control. (**D**) PKA activity and phosphorylation level of CREB were assayed after treatment with LXR-α agonist TO901317. PKA activity was assayed using a PKA ELISA kit and the phosphorylation of p38 MAPK was analyzed by Western blotting. Results were confirmed from at least three independent experiments and values represent the means ± SEM. *P*-values were obtained by the one-way ANOVA. *P < 0.05 vs. untreated control. p38, p38 MAPK; TO, TO901317; BEA, beauvericin.

To investigate the involvement of LXR-α signaling in the p38 MAPK activation, we conducted Western blot analysis for phosphorylated p38 MAPK in cells treated with the LXR-α agonist TO901317. First, we investigated the effects of TO901317 treatment on expression of the LXR-α gene. As shown in Fig. 8A, similar to the effects of beauvericin, the expression level of LXR-α gene was increased after treatment with TO901317. Next, we examined the effects of TO901317 treatment on phosphorylation of p38 MAPK. As shown in Fig. 8B, the phosphorylation level of p38 MAPK was significantly decreased by TO901317 treatment in a time-dependent manner. These results indicate that LXR-α and the p38 MAPK pathway are reciprocally related and suggest that the depigmenting effects of beauvericin are mediated through upregulation of LXR-α, which subsequently inhibits phosphorylation of p38 MAPK resulting in downregulation of melanogenic genes.

**Figure 9:**
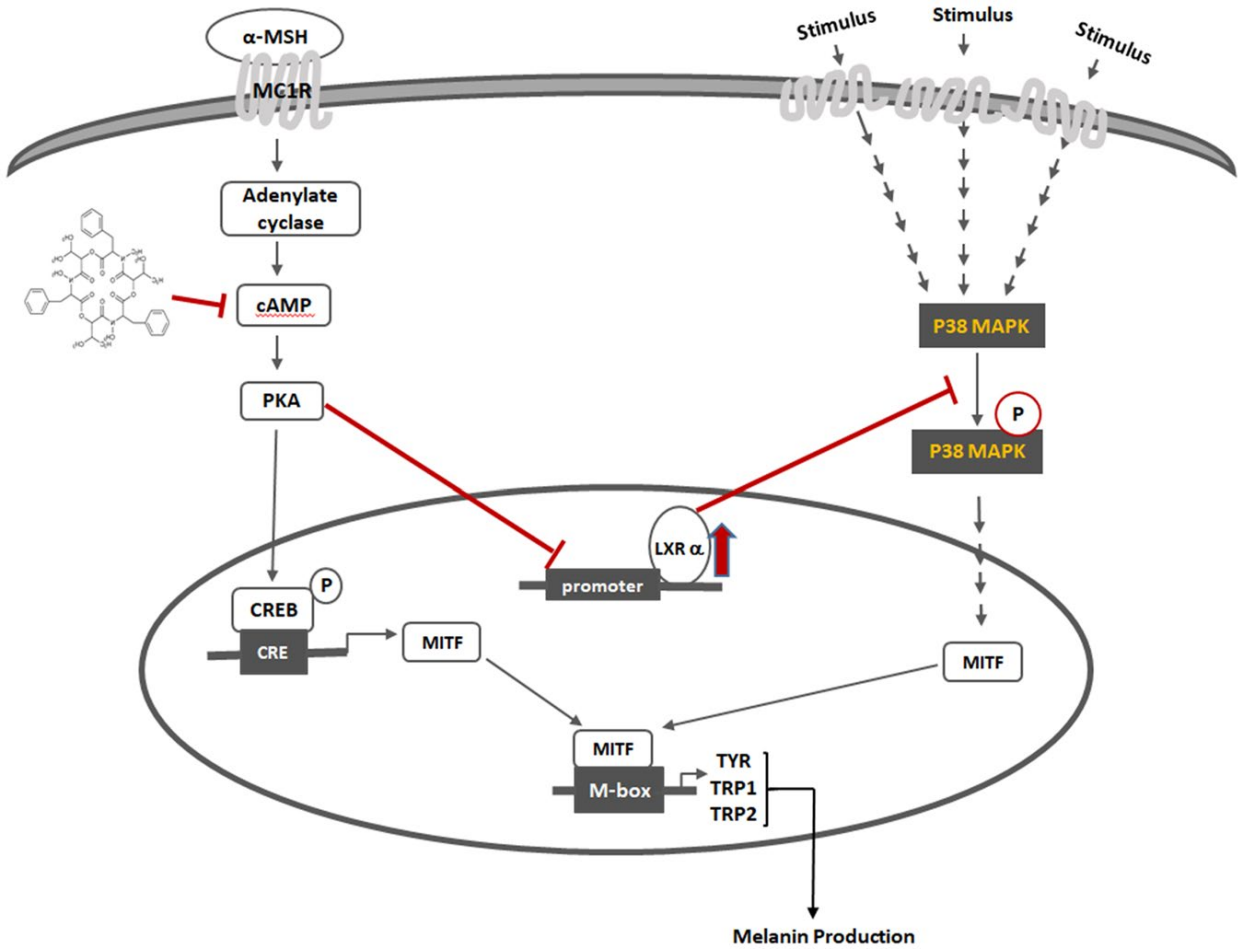
Mechanisms of beauvericin-induced anti-melanogenic effects.

To further examine the relationship of LXR-α and the cAMP-PKA-CREB signaling pathway in beauvericin-induced anti-melanogenesis activity, we investigated the effects of H89, a PKA inhibitor, on protein levels of LXR-α and phosphorylation levels of p38 MAPK. As shown in Fig. 8C, H89 increased protein levels of LXR-α but decreased the phosphorylation level of p38 MAPK. These data suggest that PKA operates upstream of LXR-α as a negative regulator, and p38 MAPK is a downstream signaling molecule of LXR-α. In addition, although H89 treatment suppressed PKA activity, TO901317 (LXR-α agonist) did not affect PKA activity (Fig. 8D). Moreover, TO901317 showed no effects on phosphorylation of CREB, a downstream molecule of PKA. Collectively, these results indicate that beauvericin inhibits cAMP/PKA/CREB signaling and LXR-α/p38 MAPK signaling, leading to inhibition of melanogenesis (Fig. 9).

## Discussion

Many researchers have investigated the mechanisms and regulation of skin pigmentation with the aim of treating or preventing hyperpigmentation skin problems^1^. However, several anti-melanogenic agents have shown adverse effects and cytotoxicity on skin cells. Therefore, it is necessary to fully understand the mechanism in order to develop more effective and safe agents.

In this study, we examined the effects of beauvericin on melanogenesis as well as the involved mechanisms. We demonstrated that beauvericin regulated melanogenesis by downregulating MITF expression through suppression of cAMP/PKA/CREB signaling and activation of LXR-α/p38 MAPK signaling. In addition, we demonstrated that LXR-α is a downstream molecule of PKA and operates upstream of p38 MAPK in beauvericin-induced anti-melanogenesis.

Several signaling pathways have been involved in pigmentation. Among the various pathways regulating melanogenesis, cyclic AMP is a key physiologic signaling molecule regulating pigmentation^4^. Cyclic AMP leads to activation of PKA, followed by phosphorylation of CREB transcription factor. CREB stimulates expression of the MITF gene, leading to an increase of melanin levels^35^. In this study, beauvericin reduced cAMP levels and suppressed CREB phosphorylation as well as PKA activity. These data suggest that the cAMP-PKA-CREB pathway may be responsible for beauvericin-induced anti-melanogenesis.

The MAPKs (p38 MAPK, ERK, JNK) and NF-κB are components of other signaling pathways for melanogenesis^1,7^. In this study, beauvericin inhibited phosphorylation of p38 MAPK, but had no effect on JNK, ERK, and NF-κB, indicating that p38 MAPK signaling is related in the depigmenting effects of beauvericin.

LXRs, consisting of LXR-α and LXR-β, are another type of melanogenic signaling molecule. These proteins are nuclear receptors that are involved in lipid and cholesterol metabolism and anti-inflammatory activities^13,15^. In particular, LXR-α is highly expressed in melanocytes from perilesional skin of patients with vitiligo and inhibits melanogenesis^11,19^. In this study, we showed that beauvericin increased expression of the LXR-α gene. Increased melanin content induced by beauvericin was attenuated by knockdown of the LXR-α gene. Furthermore, knockdown of LXR-α rescued the reduction of MITF and tyrosinase gene expression induced by beauvericin. These results suggest the involvement of LXR-α signaling in beauvericin activity.

This study demonstrated the involvement of three signaling pathways (cAMP/PKA/CREB pathway, p38 MAPK signaling, and LXR-α signaling) in the depigmenting effect of beauvericin. We therefore studied the hierarchy and relationship among these signaling pathways. Although TO901317 (LXR-α agonist) reduced phosphorylation levels of p38 MAPK, it did not affect PKA activity or the phosphorylation level of CREB, a downstream molecule of PKA. In addition, H89 (a PKA inhibitor) upregulated expression of LXR-α and suppressed phosphorylation of p38 MAPK. These data suggest that PKA operates upstream of LXR-α as a negative regulator, and p38 MAPK is a downstream signaling molecule of LXR-α. Collectively, these results indicate that the beauvericin effect is mediated by inhibiting cAMP/PKA/CREB pathway and activating LXR-α/p38 MAPK pathway, consequently leading to inhibition of melanogenesis (Fig. 8).

Recently, our group demonstrated the anti-melanogenic mechanisms of resorcinol, which is one of the phenolic components of argan oil^36^. Similar to beauvericin, resorcinol suppressed the enzymatic activity of tyrosinase. In addition, it activated the p38 MAPK pathway, leading to inhibition of melanogenesis. However, the difference was that, while resorcinol inhibited cAMP signaling, beauvericin had no effects on it. This indicates that the molecular target of beauvericin is more specific than resorcinol, suggesting the possibility that the side effects of beauvericin are not greater than those of resorcinol. In addition, it suggests that beauvericin may be selectively used as an inhibitor of p38 MAPK signaling in treating hyperpigmentation disorders.

Melanin scavenges free radicals, chelates metal cations and cellular toxins, and consumes intracellular oxygen, thus leading to hypoxia. Owing to these properties, melanin has various physiological functions^2,37,38^. Therefore, in normal melanocytes, melanin protects against ultraviolet radiation and oxidative stress. Melanin is also a marker of melanocyte differentiation and affects behavior of melanocytes and their surrounding environment. However, in melanomas, melanogenesis is not properly regulated, inducing an oxidative microenvironment with secondary mutagenic effects and producing highly immunosuppressive intermediates^39–42^. These all can potentially contribute to more aggressive cancers. In addition, melanin can cause resistance of melanoma cells to various types of therapy including radiotherapy, immunotherapy, and chemotherapy^39–42^. In this study, we demonstrated that beauvericin regulates melanogenesis in both melanoma cells and normal cells. Beauvericin has been also reported to have several beneficial properties such as insecticidal, antimicrobial, anti-tumor, and anti-inflammatory activities^21,22,31^. The results of our study suggest that beauvericin may be used as an agent to enhance the susceptibility of melanoma cells to various types of therapy as well as to treat hyperpigmentation disorders.

In conclusion, the results of this study demonstrate that beauvericin inhibits melanogenesis through inhibition of cAMP/PKA signaling, which induces upregulation of LXR-α and inhibition of p38 MAPK phosphorylation.

## Materials and Methods

### Cell culture

B16F10 cells (Cascade Biologics, Portland, OR, USA), a mouse melanoma cell line, were cultured in Dulbecco’s modified Eagle’s medium (DMEM) supplemented with 10% fetal bovine serum (FBS) and 1% antibiotics (penicillin/ streptomycin) in a humidified 5% CO_2_ atmosphere at 37 °C. Cultured human epidermal melanocytes (neonatal, moderately pigmented donor) were obtained from Cascade Biologics (Portland, OR, USA) and maintained in Medium 254 (Cascade Biologics) supplemented with Human Melanocyte Growth Supplement (HMGS) at 37 °C in a humidified atmosphere containing 95% air/5% CO_2_. For experiments, melanocytes were used at passage 2 or 5 and maintained in Medium 254 (Cascade Biologics) supplemented with HMGS.

### Assay for cell viability

The effect of beauvericin (Sigma-Aldrich, St. Louis, MO, USA) on the viability of B16F10 cells or human epidermal melanocytes was determined using cell counting kit-8 (CCK-8, Dojindo, Japan). B16F10 cells cultured in 6-well plates were treated with various concentrations (1 μM, 5 μM, 10 μM) of beauvericin and incubated at 37 °C for 24 h. CCK-8 (8 μL/well) was added to each well and the plates were incubated at 37 °C for a further 2 h. The supernatant was added to 96-well plates and the absorbance was measured at 450 nm using a microplate reader (Synergy HTX Multi-Mode Reader, Biotek, VT, USA).

### MBTH (3-methyl-2-benzothiazolinonehydrazone hydrochloride) assay for tyrosinase (dopa oxidase) activity

B16F10 cells or human epidermal melanocytes were incubated in 6-well plates and incubated at 37 °C overnight. Cells were treated with beauvericin for 24 h and then washed, harvested with PBS, and centrifuged for 5 min at 13,000 rpm. The supernatant was discarded, and the cells were lysed using a sonicator with lysis buffer (50 mM pH 6.8 sodium phosphate, 1% Triton X-100, and 1 mM phenylmethanesulfonyl fluoride [PMSF]) for 15 min. Reaction buffer (50 mM sodium phosphate pH 7.1, 2% (by vol.) N, N-dimethylformamide, 1 mM L-dopa, and 6 mM MBTH, final pH of 6.9) was added to the 96-well plate to determine the activity of tyrosinase. After incubation for 10 min at 37 °C, the amount of generated pink product was measured by absorbance at 505 nm using a microplate reader^43^.

### Assay for tyrosine hydroxylase activity

Tyrosine hydroxylase assay was performed as previously described^44^. Firstly, tetrahydrobiopterin (BH4), iron(II) sulfate, and cell extract containing tyrosine hydroxylase were mixed (mixture A) and incubated for 10 min on ice. During this incubation, tyrosine, HEPES (10 mM), and sodium periodate were mixed to make a second mixture (mixture B). A and B were combined in a 96-well plate in a 1:1 ratio to induce reaction (final concentrations: BH4 (0.25 mM), iron (2.5 μM), tyrosine (50 μM), and sodium periodate (100 μM)). Absorbance was immediately measured at 475 nm using a microplate reader. After an initial mixing for 3 s, the plate was read every 30 s for 30 min at 37 °C.

### Assay for melanin content

Cells were seeded in 6-well plates and incubated at 37 °C overnight. Cells were treated with beauvericin for 24 h and then washed, and harvested with PBS, and centrifuged for 5 min at 13,000 rpm. The supernatant was discarded and the cells were dissolved in 1 mL of a mixture of Soluene-350:water = 9:1 (v/v) by heating in a boiling water bath for 45 min as described previously^45^. The melanin content was measured by absorbance at 500 nm using a microplate reader.

### mRNA analysis for target genes

TRIzol reagent (ThermoFisher Scientific, Waltham, MA, USA) and moloney murine leukemia virus reverse transcriptase (ThermoFisher Scientific) were used to extract total RNA from cells and synthesize cDNA, respectively. An ABI7900HT Real-time PCR Instrument (Applied Biosystems, Waltham, MA, USA) was introduced for real-time RT-PCR analysis. The real-time RT-PCR analysis was conducted using TaqMan probes (Applied Biosystems) including Tyrosinase (ID: Mm00495818_m1), MITF (ID: Mm01182484_m1), TRP-1 (ID: Mm01268471_m1), TRP-2 (ID: Mm01225584_m1), hypoxanthine-guanine phosphoribosyltransferase (HPRT) (Mm00446966_m1), 18S (Mm04277571_s1), and GAPDH (ID: Mm99999915_g1). The PCR reaction was done with parameters as follows: 50 °C for 2 min, 60 °C for 30 min, and 95 °C for 5 min, and then 45 cycles of 94 °C for 20 s and 60 °C for 1 min as described previously^36^. The PCR results were normalized to the expression level of three housekeeping genes (*GAPDH*, *18S* and *HPRT*) using ABI sequence detector software version 2.0 (Applied Biosystems). Results were confirmed from at least three independent experiments.

### Small interference RNA (siRNA) transfection

ON-TARGETplus SMARTpool mouse Nr1h3 (LXR-α) siRNA (L-040649-01-0005) and ON-TARGETpuls Non-targeting pool (D-001810-10-20) were synthesized by Dharmacon Research (Lafayette, CO, USA). The cells were transfected with 25 nM of the indicated siRNAs for 48 h using DarmaFECT transfection agent (Dharmacon Research) according to the manufacturer’s instructions.

### Western blot analysis

B16F10 cells were seeded in 60-mm dishes. The cells were harvested and centrifuged for 5 min at 13,000 rpm. The supernatant was discarded and the cells were lysed with RIPA lysis buffer [25 mM Tris-HCl (pH 7.6), 150 mM NaCl, 1% NP-40, 1% sodium deoxycholate, 0.1% SDS (ThermoFisher Scientific, Waltham, MA, USA)] containing Halt protease and phosphatase inhibitor cocktail (ThermoFisher Scientific). Proteins extracted from the cells were separated by 8–10% SDS electrophoresis and transferred onto nylon membranes. The membranes were blocked with 5% skim milk for 1 h and then incubated with primary antibodies at 4 °C overnight. The membranes were washed three times in Tris-buffered saline (TBS) containing Tween 20 and probed with secondary antibodies for 1 h at room temperature. The blots were visualized using ECL Western Blotting Reagents.

### cAMP assay

The cAMP level was determined using a cAMP immunoassay kit (Cayman, Ann Arbor, MI, USA). In brief, B16F10 cells were incubated with the indicated concentration of beauvericin for 4 h. The cells were harvested and lysed in HCl (0.1 M) to suppress phosphodiesterase activity and incubated at room temperature for 30 min. The supernatants were collected in e-tubes. A constant concentration of cAMP-acetylcholinesterase (AchE) conjugate (Tracer) and the supernatant were added to a 96-well plate. After incubation at 4 °C for 18 h and washing to remove unbound cAMP, Ellman’s reagent was added to the wells to measure the activity of cAMP. The absorbance was read at 405 nm using a microplate reader. The intensity of the color, which was proportional to the amount of cAMP Tracer, was inversely proportional to the concentration of cAMP in the wells.

### Assay for β-galactosidase and luciferase reporter activities

The LXR-α promoter-luciferase reporter was generated by our group as follows. A region of the mouse LXR-α promoter from −926 to +413 (NCBI Reference Sequence: NC_000068.7) was chemically synthesized and cloned into the pGL4.11 [*luc2*P] vector (Promega Corporation, Madison, WI, USA) using two restriction enzyme sites (SpeI and XhoI). Cells were seeded in 6-well plates and incubated at 37 °C overnight. The cells were co-transfected with 1 μg LXR-α promoter-luciferase reporter and 1 μg β-galactosidase vector (Promega Corporation) using 5 μg polyethylenimine (Sigma-Aldrich, St. Louis, MO, USA) to assay for LXR-α promoter-luciferase activity. At 4 h after transfection, the cells were cultured in new medium for 24 h and then incubated in the presence of beauvericin for 24 h. β-galactosidase activity was assayed using the β-galactosidase Enzyme Assay System with Reporter Lysis Buffer (Promega Corporation). The cells were harvested with PBS and lysed with Reporter Lysis Buffer (Promega Corporation). The cells were centrifuged and the supernatants were transferred to 96-well plates to assay for β-galactosidase activity. Color development was stopped by addition of 1 M sodium carbonate to the wells and the absorbance at 420 nm was measured using a microplate reader to assay for β-galactosidase activity. Luciferase activity was assayed using the Luciferase activity Assay System (Promega Corporation). The cells were harvested with PBS and lysed with Reporter Lysis Buffer (Promega Corporation). The cells were centrifuged and the supernatants were transferred to 96-well plates. Luciferase assay substrate and Luciferase assay buffer were added to the well and luminescence was determined using a microplate reader. Activity was expressed as the ratio of LXR-α dependent firefly luciferase activity to β-galactosidase activity.

### Assay for protein kinase A (PKA)

PKA activity was evaluated using a PKA activity assay kit purchased from Enzo Life Sciences, Inc (Farmingdale, NY, USA). B16F10 cells were treated with the indicated concentration of beauvericin for 4 h. The cells were harvested and washed with PBS and lysed by incubation in lysis buffer at 4 °C for 10 min. The cell lysates were centrifuged at 15000 rpm for 15 min at 4 °C to obtain supernatant as a protein source. The protein samples and diluted ATP were added to 96-well plates which were pre-coated with substrate peptide for PKA and the plates were incubated at 30 °C for 90 min. The reaction was stopped by removing the contents from the wells. The phosphospecific substrate antibody was added to each well and the plates were incubated at 30 °C for 60 min. The wells were washed with washing buffer four times and incubated with anti-rabbit IgG for 30 min at room temperature. The wells were washed again four times with washing buffer and TMB substrate was added. The plates were incubated at 30 °C for 60 min to develop the color. Finally, stop solution 2 was added to the wells to stop the color development and the absorbance at 450 nm was measured using a microplate reader.

### Histochemical staining of reconstructed epidermis

Reconstructed human epidermis (MatTek corporation, Ashland, MA, USA) consisting of normal human-derived epidermal keratinocytes and NHEM was cultured to form a multilayer and become highly differentiated. Keratinocytes and NHEM in the reconstructed human epidermis were derived from Asian skin types. Reconstructed epidermis was incubated in medium containing beauvericin (10 μM) for 2 weeks. Compound added to the lower well of the reconstructed epidermal system could penetrate through the membrane to reach the basal cells. The medium containing beauvericin was replaced every other day. To visualize melanin pigments, the epidermis was fixed with 4% formalin in PBS, followed by Fontana–Masson staining. Briefly, the epidermal cells were washed twice with dH_2_O and then incubated with Fontana ammoniacal silver solution (American MasterTech, Lodi, CA, USA) at 37 °C for 1 h. For staining of the reconstructed epidermis, Fontana-Masson Staining Kit (American MasterTech) was used according to the manufacturer’s instructions. Quantification of Fontana-Masson staining was performed using Image J software. A total of 81 cells were analyzed per condition (three microscopic fields per condition, nine cells per microscopic field, three different experiments).

### Statistical analysis

All of the data are expressed as means ± standard error of the mean (SEM). Differences between two groups were performed using Student’s *t* test. The comparison between multiple groups was performed using the one way analysis of variance (ANOVA), and it was followed by the Tukey’s multiple-comparison test for which the GraphPad Prism (5.0) (GraphPad, La Jolla, CA, U.S.A.) was used. Statistical significance was considered when the *p* value is less than 0.05.

## Electronic supplementary material

Supplementary Information

## Data Availability

All data generated or analysed during this study are included in this published article (and its Supplementary Information files).
