# Supplementary material for: Beauvericin inhibits melanogenesis by regulating cAMP/PKA/CREB and LXR-α/p38 MAPK–mediated pathways
Source: Sci Rep. 2018 Oct 8;8:14958. doi: 10.1038/s41598-018-33352-8 (PMC6175938; doi:10.1038/s41598-018-33352-8)
Supplement: Supplementary file 1 — Supplementary Information [file 41598_2018_33352_MOESM1_ESM.pdf]

Supplementary Information

Beauvericin inhibits melanogenesis by regulating cAMP/PKA/CREB and LXR-α/p38 MAPK-mediated pathways

Seung Eun Lee[a,1], See-Hyoung Park[d,1], Sae Woong Oh[a], Ju Ah Yoo[a], Kitae Kwon[a], Se Jung Park[a], Jangsoon Kim[a], Hak Sung Lee[e], Jae Youl Cho[b,c,*], Jongsung Lee[a,c,*]

[a]*Molecular Dermatology Laboratory, Department of Integrative Biotechnology & Biocosmetics Research Center, College of Biotechnology and Bioengineering, Sungkyunkwan University, Suwon City, 16419 Gyunggi Do, Republic of Korea*

[b]*Molecular Immunology Laboratory, Department of Integrative Biotechnology, College of Biotechnology and Bioengineering, Sungkyunkwan University, Suwon City, 16419 Gyunggi Do, Republic of Korea*

[c]*Biocosmetics Research Center, College of Biotechnology and Bioengineering, Sungkyunkwan University, Suwon City, 16419 Gyunggi Do, Republic of Korea*

[d]*Department of Bio and Chemical Engineering, Hongik University, 30016 Sejong City, Republic of Korea*

[e] *Food Science R&D Center, Kolmar BNH Co., Ltd., 30003 Sejong City, Republic of Korea*

Raw Data (Fig. 2)

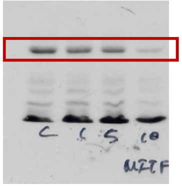

MITF

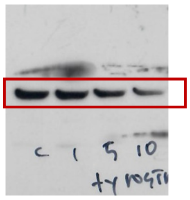

Tyrosinase

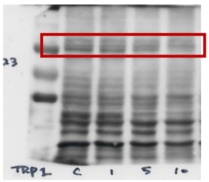

TRP1

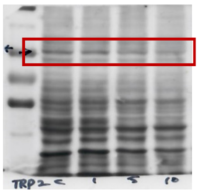

TRP2

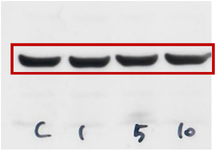

β-actin

Raw Data
(Fig. 3)

p-CREB

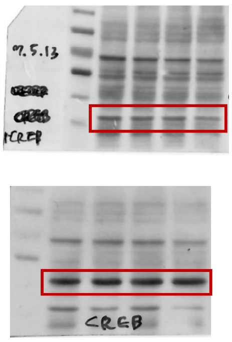

CREB

Raw Data
(Fig. 4)

p-p38 | p-ERK | p-JNK | p-NF-κB

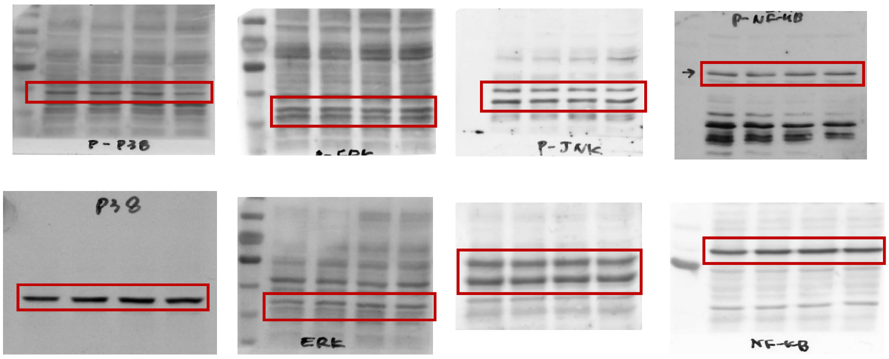

p38 | ERK | JNK | NF-κB

Raw Data
(Fig. 5)

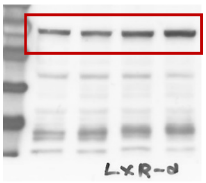

LXR-α

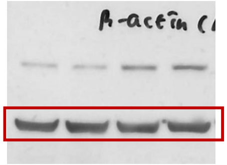

β-actin

Raw Data
(Fig. 6)

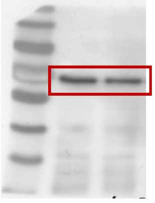

LXR-α

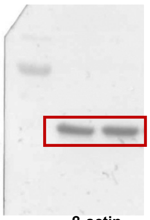

β-actin

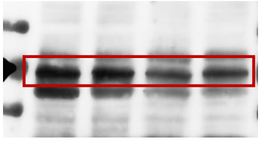

MITF

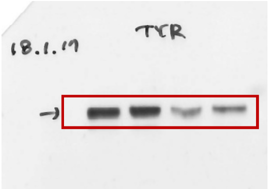

Tyrosinase

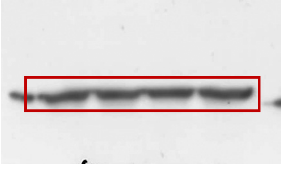

β-actin

Raw Data
(Fig. 7)

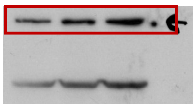

LXR-α

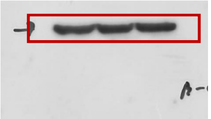

β-actin

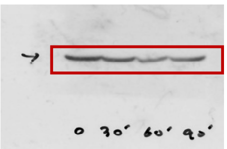

p-p38

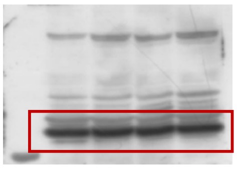

p38

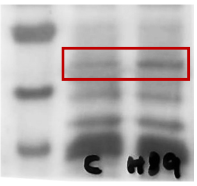

LXR-α

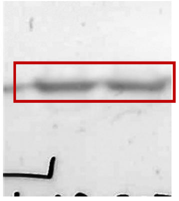

β-actin

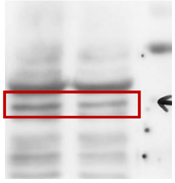

p-p38

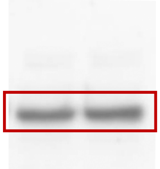

p38

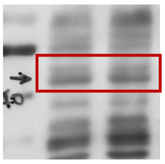

p-CREB

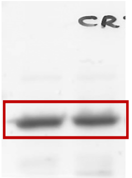

CREB
